# Guest Editors’ Introduction: The role of policy in reducing malnutrition in sub-Saharan Africa

**DOI:** 10.1016/j.foodpol.2022.102378

**Published:** 2022-11

**Authors:** Théophile T. Azomahou, Raouf Boucekkine, Harounan Kazianga, Mark Korir, Njuguna Ndung'u

**Affiliations:** a African Economic Research Consortium, Nairobi, Kenya; b School of Economics and Institute for Advanced Study, Aix-Marseille University, France; c Spears School of Business, Oklahoma State University, United States

**Keywords:** Agricultural, food and nutrition policy, Nutrition outcomes, Food security, Dietary diversity, Malnutrition

## Abstract

Sub-Saharan African countries experience various market failures and other constraints in food production, marketing, and food consumption. Consequently, sub-Saharan Africa governments have put in place a myriad of policies to counter these failures. Agricultural, food and nutrition policies address nutrition outcomes, such as hunger, undernourishment, wasting, stunting, child mortality, inadequate food consumption, food insecurity, and volatile food prices, thus improve nutrition outcomes among the population. However, malnutrition persists among the population in the region. To mitigate this challenge, informed, evidence-based policy development and implementation by policy practitioners is of essence. The solutions to the double burden of undernutrition and obesity cut across the agriculture, rural development, and public health sectors. This essay introduces twenty papers of this Special Issue of the Food Policy journal which analyzes 8 policy domains, contributes to the debate on the linkages and pathways through which policies influence food security, nutrition outcomes, and related indicators and points to policy directions in these domains.

## 1 Introduction

The world over, malnutrition continues to be a pressing problem. According to Marcello et al. (2022), worldwide prevalence of undernourishment and food insecurity has been steadily increasing. Unhealthy diets are a major cause of non-communicable diseases which contribute to an increase in overweight, obesity and malnutrition. Malnutrition is rampant in sub–Saharan African countries, with infants lacking access to adequate nutritious foods with cases of chronic malnutrition (Faria, 2022).

Sub-Saharan African countries experience various market failures and other constraints in food production, marketing, and consumption of nutritious food. For example, the market has been unable to deliver farm inputs, particularly fertilizers to smallholder farmers at prices they can afford. Similarly, the less endowed populations have inadequate access to nutritious foodstuffs. Consequently, many policies around agricultural value chains have been put in place to counter these failures. However, malnutrition among the population in the region is still high. In Ethiopia, for example, the occurrence of growth retardation among children under-5 years is 38 %, underweight 24 % and wasting at 10 % (Kasahun et al., 2020). To mitigate this challenge, informed, evidence-based policy development and implementation by policy practitioners is of essence.

Agricultural, food and nutrition policies address nutrition outcomes, such as hunger, undernourishment, wastage, stunting and child mortality, inadequate food consumption, food insecurity, and volatile food prices, thus improve nutrition among the population. Agricultural trade policies governing imports and exports, and infrastructure policies affect trade, and hence availability of food. Similarly, policies, such as research and extension, social safety nets, consumption subsidies, etc., raise consumer purchasing power, and thus enable consumers to access food products for enhanced nutrition (HLPE, 2014).

While researching on better nutrition and defining future directions in Europe, Bredaa et al (2020) note that, while significant progress has been made in some areas of public health nutrition, European countries need more ambitious policies with appropriate breadth and depth to achieve global noncommunicable disease (NCD) targets. Sub-Saharan Africa requires even more ambitious policies to tackle the double burden of undernutrition and obesity.

The current trend of research is towards a multidisciplinary and interdisciplinary approach to current problems. In the recent past, several consortia have emerged to research in agriculture, nutrition, and health for informed nutrition policy. Some examples include Agriculture, Nutrition and Health (ANH) Academy (https://www.anh-academy.org/), Food Environment Research Network (FERN), FAO- Monitoring and Analyzing Food and Agricultural Policies (MAFAP), Innovative Methods and Metrics for Agriculture and Nutrition Actions (IMMANA), and International Dietary Data Expansion (INDDEX) Project, among others. According to Fanzo (2014), the solutions to the double burden of undernutrition and obesity cut across sectors, engaging practitioners and experts across agriculture, rural development, and public health. Consequently, improvements can be driven by resilient food system approaches to ensure better utilization of food, dietary diversity, and quality.

This Special Issue presents research on how various agricultural and food policies in eight policy domains influence food security and various nutrition outcome indicators in sub-Saharan Africa (Table 1). The research papers contribute to the debate on the linkages and pathways through which policies influence food security, nutrition outcomes, and related indicators and point to future frontier research and policy direction.

**Table 1:** Policy domains and food security, nutrition outcome and related indicators.

*No.*	*Policy Domain*	*Author(s)*	*Variables of Interest*	*Outcomes of Interest*
1	Agricultural extension	Sibhatu, K. T. *et. al*.	Nutrition education	Months of Adequate Household Food Provisions (MAHFP); Food Consumption Score (FCS) and Household Dietary Diversity Score (HDDS)
		Bambio, Y. *et. al*.	Technology adoption	Consumption and food security index
2	Capital and productivity	Nguyen-Anh, T. *et. al.*	Human capital	Calorie intake
		Kinda, S. R. *et. al.*	Land rush	Food production
		Villacis, A.H. *et. al.*	Value of agricultural productivity	Less preferred foods, variety of food eaten, and portion size at mealtimes
3	Input subsidy	Matita, M.	Legume seed subsidy	Dietary diversity
		Mwale, M. *et. al.*	Fertilizer (basal and top dressing), maize seed, flexible voucher (for maize seed or legume)	Height-for-age
		Khonje M.G. *et. al.*	Legume seed subsidy	Calorie, Vit A and Zinc intake; Weight-for-height; Dietary diversity
		Kuntashula, E.	Farm input subsidy (hybrid maize seed and fertilizer)	Production diversity and dietary diversity
4	Markets and commercialization	Nkegbe, P.K. *et. al.*	Transaction costs	Food Consumption Score
		Jema, H.	Marketed surplus	Stunting
		Kilimani, N.	Crop commercialization	Nutrient intake
		Usman, M. A.	Market access	Dietary diversity
		Chegere and Kauky	Dietary diversity, nutritional status	Agricultural Commercialization Index (AIC)
		Wondimagegn, T.	Crop diversification	Child growth
5	Output price policy	Schneider, K. R.	Food prices	Cost of diets
6	Climate change	Van der Merwe *et. al.*	Monthly maximum average temperature, total monthly precipitation	Stunting and underweight
7	Nutrition	Azomahou, T. T. *et. al.*	Nutritional Status	Body Mass Index (BMI)
		Malacarne and Paul	Agricultural management practices, drought	Food security, dietary diversity
8	Government spending	Hendriks S. *et. al*.	Public agriculture investment	Dietary energy supply

## 2 Meeting nutrition policy needs: Evidence from this SI

This Special Issue contributes to the evidence that agricultural, food and nutrition policies have bearing on food security and nutrition outcomes such as malnutrition, stunting, obesity, wasting and related nutrition outcome indicators. It also provides evidence on the linkages and pathways between policy interventions and food security indicators, such as food production diversity and food consumption diversity. According to Kadiyala et al, (2014), existing studies are rich in weak associations between policies and nutrition outcomes. This Special Issue provides the evidence on the impact of agricultural and food policies on food security, nutrition outcomes and related indicators. Further, it illuminates on policy implications of these findings and contributes to the debate around agricultural policies, nutrition, and health. Here below are the specific policy domains and the respective food security, nutrition outcome and related indicators covered. A total of 20 papers clustered into eight policy domains are presented.

### 2.1 Agricultural extension policies

The important role agriculture plays in African economies and livelihoods, and the strong linkages that agriculture forges with other sectors cannot be overemphasized (Sarpong, 2021). African governments, therefore, have established agricultural extension services to offer technical knowledge on production, marketing, and nutrition education, among other subjects to farming communities to improve productivity and well-being. Sibhatu et al., (2022) shows that the Smallholder Productivity Promotion extension programme in Zambia increased productivity, income, higher dietary diversity, and food security. The extension programme in Ghana, Mali and Senegal, raised the adoption of improved seeds by 0.20 percentage points and increased productivity and income of the treated households by 10 % and 17 %, respectively. However, there were no effects on consumption and food security (Bambio et al., 2022).

### 2.2 Capital and agricultural productivity

Improving agricultural productivity is a priority concern in promoting the sustainable development of agriculture in developing countries. This requires an optimal combination of various capital and productive inputs such as human, social, land and farm inputs, among others. According to Nguyen-Anh et al. (2022), social capital is the most critical factor among the three groups of capitals (human, natural and social) in promoting farming productivity in SSA.

Kinda et al. (2022) document that a steady increase in commodity prices in the first decade of the 2000 s, and the financial, food and economic crisis have triggered an unprecedented rush for farmland, especially in developing countries. They investigate the effect of a land rush on food security during the period 2000–2018 in 32 SSA countries and find out that land rush has adverse effects on the production of cereals and increases malnutrition.

Villacis et al. (2022) investigates the impact of agricultural productivity on experience-based measures of food security among Nigerian households and find out that an increase in agricultural productivity decreases the probability of a household relying on less preferred foods.

### 2.3 Input subsidy policies

Farm input subsidy programmes aimed at promoting both agricultural production diversity and dietary diversity as the gateways for improving household nutritional status, have been a major agricultural policy thrust in most sub-Saharan Africa (Kuntashula, 2022). Input subsidies aim at increased access by producers to technology and high-yielding varieties. In Malawi, Matita (2022) shows that access to legume seed coupons and their redemption is positively associated with diversified diets while the Farm Inputs Subsidy Program (FISP) positively contributed to production diversity (crop count, agricultural income diversity and household income diversity) (Kuntashula, 2022). Mwale et al. (2022) found out that while subsidy programs are aimed at enhancing food security, they only improve nutrition outcomes that are sensitive to intervention typically in the short-run, weight for-age and weight-for-height, but not height-for-age.

### 2.4 Markets and agricultural commercialization

Food marketing and functioning of food markets is a major contributor to high rates of nutrition challenges like obesity and diet-related disease among children. According to Alonso (2021), informal markets are one of the primary sources of food and nutrition for poor people throughout the developing world. Yet, for too long, these markets have been overlooked, underappreciated, and side-lined. These markets, which range from street-side food vendors to small-scale retailers, offer a range of benefits to both buyers and sellers across the developing world in terms of health, nutrition, and livelihoods. With more investment and research, informal markets can become a greater force for the good of the most vulnerable in low- and middle-income countries and assume a more significant positive role in the food systems of the future.

Usman (2022) shows that households located closer to market centers spend more on total household consumption expenditure, consume more diverse diets, and are less food insecure than households located further away from markets. In eastern Africa, the average consumption score of protein-rich foods consumed is 4.48 and the consumption score of vitamin A-rich foods is 4.82 and that the intake of iron-rich foods has the lowest average (Nkegbe and Mumin, 2022). In Ethiopia, Jema (2022) reveals that the average levels of underweight and wasting increase with lower and higher intensities of commercialization, but decrease with medium commercialization intensities, with the overall significant beneficial effects. Kilimani (2022) examines the channels through which crop commercialization influences household nutrient intake. He finds that commercialization mainly affects nutrient intake through the resulting crop income and concludes that interventions geared towards agricultural commercialization are beneficial to household nutrition via income generation. Further, subsidizing legume seeds increased area planted with legume crops, groundnut yield, production and dietary diversity, and consumption of micronutrients, vitamin A and zinc. It was also found out that subsidizing legume seed was positively correlated with child weight-for-age Z-score (Khonje et al., 2022).

Chegere and Kauky (2022) analyze the linkages between agricultural commercialization, dietary diversity and nutritional status in Tanzania and find out that agricultural diversity has a significant effect on dietary diversity for the lower-income groups. Also, both commercialisation and household dietary diversity have an insignificant effect on child stunting, wasting, and underweight even for the lower-income group. Wondimagegn (2022) documents that crop diversification has a positive but small impact on child growth and that the positive effects are more pronounced in areas with limited access to markets.

### 2.5 Output price policy

According to von Braun (2012), the food price problem is far-reaching with wide and interrelated impacts and highly complex and dynamic price formation mechanism. Given that policy actions are politically and economically sensitive, this situation calls for continuous and comprehensive assessments of the problem to provide timely and evidence-based knowledge for policy makers. While demonstrating how food prices, and the availability of food items can be used to diagnose how well a food system facilitates access to nutrient-adequate diets, Schneider (2022) found out that larger households have more diverse nutrient needs and face a higher cost for 1000 calories of a sufficiently nutrient dense diet. At the prevailing prices from January 2013–July 2017, the diet was available in 60 % of all household-months at an average cost of $2.32/person/day.

### 2.6 Nutrition policy

Despite efforts by governments and development agencies, malnutrition persists among the sub-Saharan Africa population. For instance, Faria (2022) notes that Rwanda had the most acute malnutrition, with 38.3 % of infants lacking access to adequate nutrition while 36.8 % of children aged 0–4 years suffered chronic malnutrition in Ethiopia. Azomahou et al (2022) analyze transition and persistence patterns of the Double Burden of Malnutrition (DBM) and Overweight or Obesity (OVOB) and find that DBM is a transitory phenomenon as most double burden households do not remain so in subsequent surveys and OVOB is persistent at the individual level.

Malacarne and Paul (2022) investigate the associations of nutritional outcomes and agricultural management practices under drought risk and show that drought has significant consequences on food security and dietary diversity. They find out that these consequences are evident even for households using improved management practices, such as improved seed, chemical fertilizer, and production diversity.

### 2.7 Other policies

Van der Merwe et al. (2022) investigate the effect of climate change (changes in the monthly maximum average near-surface temperature and total monthly precipitation) on children’s health outcomes, particularly stunting and underweight, in Nigeria. They find out that a rise in temperature is associated with higher levels of stunting, and more so in rural areas. They note that improvements in public infrastructure, as well as aid promoting climate smart agricultural practices, can also play an important role in reducing the effect of climate change on child malnutrition.

Hendriks et al (2022) assess the effect of public expenditure on food security in nine countries in the Economic Community of West African States (ECOWAS) region. They find that, despite improvement in public agricultural expenditure, this has not translated to great improvement in food security as was desired. However, a unit increase in public agriculture expenditure was associated with a 0.2 % reduction in undernourishment and improved average dietary energy supply adequacy between 2000 and 2016.

## 3 Conclusions

Nineteen papers analyzing the impact of various agricultural, food, nutrition, and related policies are presented. Various pathways of impact between the policy variables of interest, such as input subsidy, agricultural extension, market access and commercialization, capital, and agricultural productivity, among others are showcased. We conclude that agricultural, food and nutrition policies have the potential to mitigate food inaccessibility, insecurity, and malnutrition. However, not all rural households are able to access a nutritionally adequate diet. Even when available, the cost per capita is beyond reach. Given the potential of agricultural extension, it is important to include nutrition education interventions in agricultural extension programs.

Seed Legume Subsidy is shown to contribute to production diversity, dietary diversity, vitamin consumption and weight for age. A policy of subsidizing seed legumes should therefore be pursued by governments and development agencies in addressing malnutrition in smallholder farm settings. Interventions geared towards agricultural commercialization are beneficial to household nutrition via income generation. To increase food security, African governments should reform land acquisition agreements to promote investment and production of crops for domestic consumption. Further, investing in accessible market development and rural infrastructure to link smallholder farmers to markets is essential for improving household dietary diversity and food security.

Recognizing the multifaceted nature of food security and nutrition, an intervention option should be implemented together with the other intervention options, for larger impact on food security and nutrition.

## 4 Disclaimer

The views expressed in this publication are those of the authors and do not necessarily reflect the views or policies of the African Economic Research Consortium (AERC), Aix-Marseille University or Oklahoma State University.

## Declaration of Competing Interest

The authors declare that they have no known competing financial interests or personal relationships that could have appeared to influence the work reported in this paper.
